# Serum YKL-40 Independently Predicts Outcome after Transcatheter Arterial Chemoembolization of Hepatocellular Carcinoma

**DOI:** 10.1371/journal.pone.0044648

**Published:** 2012-09-06

**Authors:** Cheng-Bao Zhu, Can Wang, Li-Li Chen, Guo-Liang Ma, Shi-Cai Zhang, Liang Su, Jian-Jun Tian, Zhong-Tao Gai

**Affiliations:** 1 Department of Clinical Laboratory, Jinan Infectious Disease Hospital, Shandong University, Jinan, Shandong, People’s Republic of China; 2 Department of Hepatology, Jinan Infectious Disease Hospital, Shandong University, Jinan, Shandong, People’s Republic of China; 3 Department of Traditonal Chinese Medicine Ophthalmology, The Second People's Hospital of Jinan, Jinan, Shandong, People’s Republic of China; 4 Department of Clinical Laboratory, the People's Hospital of Laiwu, Laiwu, Shandong, People’s Republic of China; 5 Department of Clinical Laboratory, Tumour Hospital of Taian, Taian, Shandong, People’s Republic of China; University of Colorado School of Medicine, United States of America

## Abstract

**Background:**

Transcatheter arterial chemoembolization (TACE) is the most widely used treatment option for unresectable hepatocellular carcinoma (HCC). Elevated serum YKL-40 level has been shown to predict poor prognosis in HCC patients undergoing resection. This study was designed to validate the prognostic significance of serum YKL-40 in patients with HCC undergoing TACE treatment.

**Methods:**

Serum YKL-40 level was determined by enzyme-linked immunosorbent assay. Overall survival (OS) was evaluated with the Kaplan-Meier method and compared by the log-rank test. Multivariate study with Cox proportional hazard model was used to evaluate independent prognostic variables of OS.

**Results:**

The median pretreatment serum YKL-40 in HCC patients with was significantly higher than that in healthy controls *(P*<0.001). The YKL-40 could predict survival precisely either in a dichotomized or continuous fashion (*P*<0.001 and *P* = 0.001, respectively). Multivariate Cox regression analysis indicated that serum YKL-40 was an independent prognostic factor for OS in HCC patients (*P* = 0.001). In further stratified analyses, YKL-40 could discriminate the outcomes of patients with low and high alpha-fetoprotein (AFP) level (*P* = 0.006 and 0.016, respectively). Furthermore, the combination of serum YKL-40 and AFP had more capacity to predict patients’ outcomes.

**Conclusions:**

Serum YKL-40 was demonstrated to be an independent prognostic biomarker in HCC patients treated with TACE. Our results need confirmation in an independent study.

## Introduction

Hepatocellular carcinoma (HCC) is the sixth most common malignancy and third most deadly carcinoma in the world [1]. Hepatectomy and liver transplantation are currently considered to be the most effective procedure for HCC in suitable candidates [2], however, they are only suitable for 20% to 35% of patients with HCC because of poor hepatic reserve or severe organ shortage for transplantation [3], [4]. Although many non-operative treatment modalities for HCC are available, the most widely used treatment option for unresectable HCC is transcatheter arterial chemoembolization (TACE), where iodized oil mixed with anticancer agents and an embolic material is administered through the hepatic artery, and it has recently been shown to improve survival in comparison to best supportive care [5]–[7]. Recent studies have shown that several parameters like tumor size, portal invasion, alpha fetoprotein (AFP), Child-Pugh score, bilirubin, ascites, performance status and therapy response were independent predictors of survival after TACE [8]. However, these parameters are not accepted in routine clinical practice for estimation of prognosis. As a result, the prognostic function of simple serum biochemical markers that are easily obtained at outpatient clinics at a low cost is still attractive.

YKL-40 (CHI3L1) is a member of mammalian chitinase-like proteins without chitinase activity [9]. Although the biologic function of YKL-40 is not exactly known, it is suggested that this protein participates in inflammation, cell proliferation, differentiation, protection against apoptosis, stimulation of angiogenesis and regulation of extracellular tissue remodeling [10]. Microarray gene analyses has identified the human YKL-40 gene as one of the most differentially expressed genes in liver tissue [11], and overexpression of YKL-40 has been found in primary and metastatic HCC biopsies, also in primary and metastatic HCC cell lines(H2-P and H2-M) [12]. Furthermore, YKL-40 is reported to be highly expressed in HCC at the molecular, cellular and tissue levels [13]. All these studies confirmed the aberrant expression of YKL-40 in HCC, which supported that YKL-40 may play an important role in cancer cell proliferation and invasiveness.

Elevated serum YKL-40 levels were found in several primary and metastatic malignancies, and serum YKL-40 may serve as a valuable and independent prognostic biomarker of short survival [14]. Previous study has revealed that elevated YKL-40 level predicts the increased risk of gastrointestinal cancer and decreased survival after cancer diagnosis in general population [15]. A recent study by our group has demonstrated that serum YKL-40 was an independent prognostic factor for OS and RFS in HCC patients undergoing curative resection and serial monitoring of serum YKL-40 after curative resection may provide additional prognostic information [16]. However, the prognostic value of serum YKL-40 in HCC patients treated with non-operative therapy remains unclear. Hence, we conducted a prospective study to test the hypothesis that elevated serum YKL-40 in pretreatment serum samples predict poor prognosis in HCC patients undergoing TACE treatment.

## Materials and Methods

### Patients

Between February 2004 and December 2006, 212 HCC patients underwent TACE treatment at Jinan Infectious Disease Hospital, Shandong University (Shandong, China) were enrolled in this retrospective study. The diagnosis of HCC was confirmed histologically by needle biopsy or based on the findings of typical radiological features in at least two image examinations including ultrasonography, contrastenhanced computed tomography (CT), and magnetic resonance imaging (MRI). All TACE procedures were performed by a multidisciplinary team composed of hepatobiliary surgeons and interventional radiologists. Inclusion criteria for TACE treatment include disease multifocality, early vascular invasion, decompensated liver disease, or poor performance status. Exclusion criteria include patients whose main trunk of the portal vein was completely obstructed by a tumor thrombus or patients who underwent surgical resection after TACE. The study was approved by the ethics committee of Jinan Infectious Disease Hospital, Shandong University, and written informed consent was obtained from each patient.

### TACE Procedures

All TACE therapies were performed under angiographic control (Multistar TOP and Axiom Artis dTA, Siemens, Munich, Germany) and under local anesthesia. According to Seldinger’s technique of arterial embolization, a 5-F catheter was inserted via the right femoral artery, hepatic arteriography and portovenography through the superior mesenteric artery and celiac arteriography were performed to define the size and location of the tumor nodules. A mixture of lipiodol and chemotherapeutic drug (40–60 mg doxorubicin, 50–100 mg cisplatin or 10–20 mg mitomycin C, 2–30 mL iodized oil) was injected into the hepatic artery until stasis within the tumor vessels occurred, the feeding arteries to the tumors were then embolized with gelatin sponge particles. The extent of embolization was determined depending on the number and location of the tumors, vascularity, and fluoroscopic findings.

### Patient Follow-up

Liver imaging was performed by CT/MRI images within 1 month after TACE to evaluate the therapeutic efficacy. For the patients without complete tumor response (defined as no artery-phase enhancement in CT or MRI), repeat TACE procedures were performed on demand based on tumor response and patient health. TACE was performed 1–5 times (1 time for 165, 2 times for 20 and ≥3 times for 27 patients).

### Healthy Controls

The reference range of serum YKL-40 was determined in 100 healthy individuals (43 women and 57men; median age, 46 years, range, 18–71 years). Serum YKL-40 did not depend on gender, but it increased with age [17]. Therefore, a normal reference range was calculated as described by Royston on the log transformed serum YKL-40 values of the healthy controls after adjusting for age, and the 95th percentile was chosen as the cut-off value [18].

### Sample Collection and Assay of Serum YKL-40 Level

Serum samples were collected before the first TACE session and stored at –80°C until analysis of YKL-40. The levels of serum YKL-40 were quantified using a commercially available ELISA kit (Quidel Corporation, Santa Clara, CA) according to the manufacturer's instructions. The sensitivity of the assay for YKL-40 was 10µg/L, and the intra-assay and inter-assay coefficient variations of YKL-40 ELISA kit were <3.6% and <3.7%, respectively.

### Statistical Analysis

Continuous data were expressed as median and range. The chi-square test or Fisher’s exact test was used to analyze the relation between the continuous data and clinical characteristics of the patients. Regression analysis was carried out for the age correction of YKL-40 in healthy controls. Overall survival (OS) was defined as the time from the first TACE session to death. All data on disease status and duration of survival were updated by December 31, 2011. Overall survival curves were estimated by the Kaplan-Meier method and compared by the log rank test. Prognostic relevance of each variable to OS was analyzed using the Cox proportional hazards regression model. *P* value <0.05 was considered as statistically significant. All statistical analyses were performed by SAS software version 9.1 (SAS Institute, Cary, NC).

## Results

### Patient Characteristics

The patients’ characteristics are summarized in Table 1. Of the 212 patients, 135 were male and 77 female, and the median age was 53 years (range 32–79 years). The etiology for HCC was hepatitis B virus (HBV) in 163 patients, hepatitis C virus (HCV) in 36 patients, coinfection of the two hepatitis viruses in 6 patients and unknown in 7 patients. The Child- Pugh class was A in 162, B in43 and C in 7 patients. The median diameter of largest tumor was 3 cm (range 0.6–7 cm) and the numbers of tumors was in 1–5. Vein invasion evaluated on imaging findings (microvessel invasion or macrovessel invasion) was present in 56 patients.

**Table 1:** Associations between serum YKL-40 and clinical characteristics in 212 HCC patients undergoing TACE treatment.

Characteristic		Serum YKL-40 levels		*P*
		Normal	Elevated	
Age (years)				
	≤53 (n = 111)	49	62	
	>53(n = 101)	28	73	0.013
Sex				
	Male (n = 135)	51	84	
	Female (n = 77)	26	51	0.559
Etiology				
	HBV (n = 163)	56	107	
	HCV (n = 36)	18	18	0.224
	HBV, HCV (n = 6)	1	5	
	Others (n = 7)	2	5	
Child-Pugh class				
	A (n = 162)	61	101	
	B (n = 43)	12	31	0.252
	C(n = 7)	4	3	
Serum AFP (ng/mL)				
	≤20(n = 96)	43	53	
	>20(n = 116)	34	82	0.020
Tumor size (cm)				
	≤2 (n = 92)	36	56	
	>2 (n = 130)	41	79	0.456
Tumor number				
	Single (n = 149)	56	93	
	Multiple (n = 63)	21	42	0.556
Vein invasion^a^				
	No (n = 155)	59	96	
	Yes (n = 56)	18	38	0.430
Number of TACE sessions				
	Single (n = 165)	58	107	
	Multiple (n = 47)	19	28	0.507

a Evaluated on imaging findings.

HBV, hepatitis B virus; HCV, hepatitis C virus; AFP, alpha-fetoprotein.

### Associations between Serum YKL-40 and Patients Characteristics

The median pretreatment serum YKL-40 levels in 212 patients with HCC was 185µg/L (range, 12–1423), which was significantly higher than the level in 100 healthy controls after correction for age (median 51µg/L, range, 11–184 µg/L, 95th percentile is 106 µg/L) *(P*<0.001). A serum YKL-40 above the age-adjusted 95th percentile of the healthy controls was seen in 64% (135/212) of the patients. The 95th percentile of the age-adjusted YKL-40 values in healthy controls was used as the cut-off value, and then serum YKL-40 is dichotomized in normal versus elevated YKL-40 levels for the following analyses.

Table 1 shows the association between serum YKL-40 levels and clinical characteristics. Serum YKL-40 was associated with age (*P* = 0.013) and serum AFP (*P* = 0.020), but not with the other characteristics such as gender, etiology, Child-Pugh class, tumor size, vein invasion and number of TACE sessions.

### Overall Survival Results after TACE

At the time of analysis, 158 patients (74.5%) died from systemic failure (74 cases), hepatic encephalopathy (43 cases), gastrointestinal hemorrhage (30 cases), and other causes (11 cases). The cumulative 1-year, 2-years, 3-years, and 5-years OS rates were 57.1, 39.6, 21.6, and 5.9%, respectively, and the median OS was 13.5 months.

### Prognostic Significance of Serum YKL-40 Level

**Figure 1:**
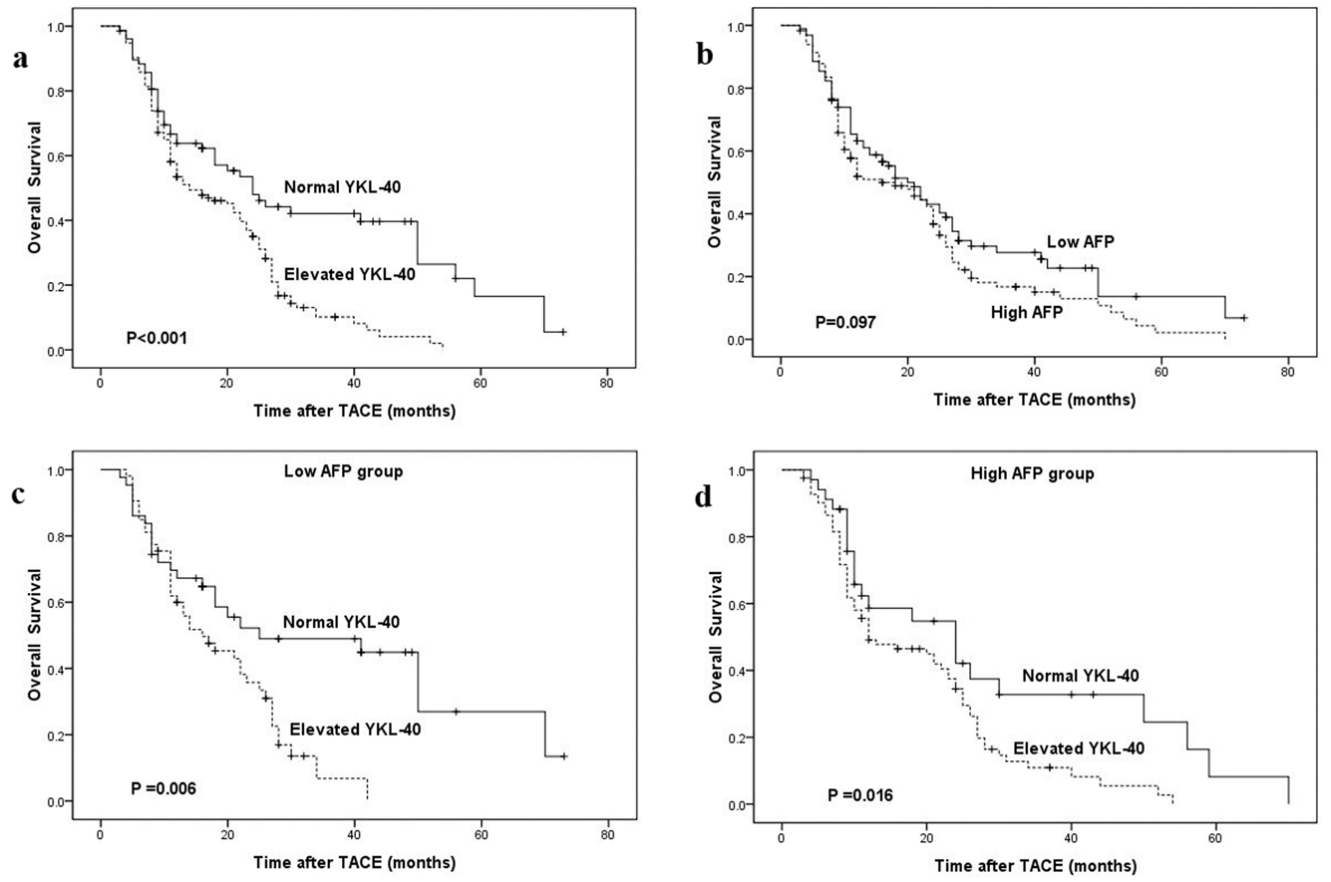
Kaplan-Meier survival curves stratified by serum YKL-40 and AFP. **a** OS curve classified by YKL-40 in all patients (n = 212). **b** OS curve classified by AFP in all patients (n = 212). **c** OS curve classified by YKL-40 in low AFP group (n = 96). **d** OS curve classified by YKL-40 in high AFP group (n = 116).

The Kaplan-Meier estimates of overall survival stratified by serum YKL-40 dichotomized are shown in Figure 1a. Patients with elevated serum YKL-40 had significantly shorter overall survival than patients with normal serum YKL-40 (median 12 months vs.16 months; *P*<0.001, log-rank test). When serum YKL-40 was log transformed as continuous variable, serum YKL-40 log transformed was significantly associated with OS [hazard ratio (HR) = 1.811, 95% confidence interval (CI): 1.273–2.575, *P* = 0.001].

In addition, by Kaplan-Meier analysis and log-rank test, we observed that overall survival was not associated with serum AFP level (*P* = 0.097, Fig. 1b). Strikingly, in the stratified analyses according to AFP level, serum YKL-40 level could further discriminate the outcomes of patients with low and high AFP level (*P* = 0.006 and 0.016, respectively; Fig. 1c-d). Patients with elevated YKL-40 had poorer clinical outcomes than those with normal YKL-40, no matter what AFP they had.

**Figure 2:**
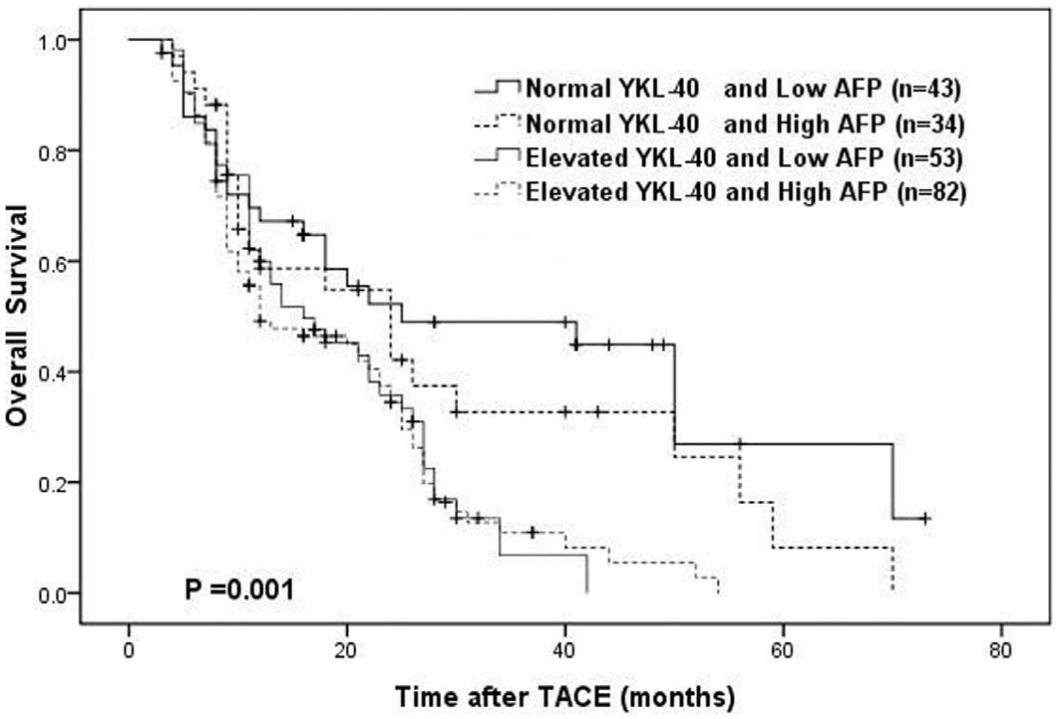
OS curves of the combination of serum YKL-40 and AFP level.

When serum YKL-40 and AFP were taken into consideration together, patients were classified into four groups according to their serum YKL-40 and AFP level: normal YKL-40 and low AFP level group (n = 43); normal YKL-40 and high AFP level group (n = 34); elevated YKL-40 and low AFP level group (n = 53); elevated YKL-40 and high AFP level group (n = 82). Fig. 2 shows the overall survival curve of these four groups. The OS rates were significantly higher in the normal YKL-40 and low AFP group compared with the elevated YKL-40 and/or high AFP level group (*P* = 0.001). The 3-year OS rates in normal YKL-40 and low AFP group was 49.0%, which was significantly higher than those of elevated YKL-40 and high AFP level group (10.9%, *P*<0.001).

### Factors Associated with Overall Survival by Univariate and Multivariate Analyses

Age, sex, etiology, Child-Pugh class, tumor size, tumor numbers, serum AFP, serum YKL-40, vein invasion, and number of TACE sessions were enclosed in the univariate analysis. The results showed that serum YKL-40 dichotomized (*P*<0.001), tumor size (*P* = 0.001), vein invasion (*P* = 0.006), and number of TACE sessions (*P* = 0.009) were significant prognosis factors associated with OS (Table 2). All significant covariates in the univariate analyses were entered into a multivariate Cox analysis. SerumYKL-40 (HR = 1.809, 95%CI: 1.259–2.601, *P* = 0.001) together with tumor size (HR = 1.486, 95%CI: 1.069–2.067, *P* = 0.018) emerged as independent prognostic factors for overall survival (Table 2).

**Table 2:** Univariate and multivariate analyses of prognostic factors associated with overall survival.

Covariates	Univariate analyses			Multivariate analyses		
	HR	95% CI	*P*	HR	95% CI	*P*
Age (≤53 vs. >53 years)	0.992	0.723–1.362	0.962			
Sex (female vs. male)	0.927	0.667–1.289	0.654			
Etiology (HBV vs. HCV)	1.124	0.762–1.658	0.554			
Child-Pugh class (Class A vs. Class B)	1.190	0.796–1.779	0.397			
Serum AFP (≤20 vs. >20 ng/mL)	1.297	0.944–1.780	0.109			
Tumor size (≤2 vs. >2 cm )	1.693	1.225–2.339	0.001	1.486	1.069–2.067	0.018
Tumor number (single vs. multiple)	1.228	0.880–1.714	0.227			
Vein invasion^a^(no vs. Yes)	1.609	1.150–2.251	0.006	1.400	0.996–1.96	0.053
Number of TACE sessions(multiple vs. single)	0.589	0.396–0.875	0.009	0.713	0.475–1.071	0.104
Serum YKL-40 (normal vs. elevated)	1.973	1.372–2.838	<0.001	1.809	1.259–2.601	0.001

a Evaluated on imaging findings.

HBV, hepatitis B virus; HCV, hepatitis C virus; AFP, alpha-fetoprotein.

### Serum YKL-40 and AFP

**Figure 3:**
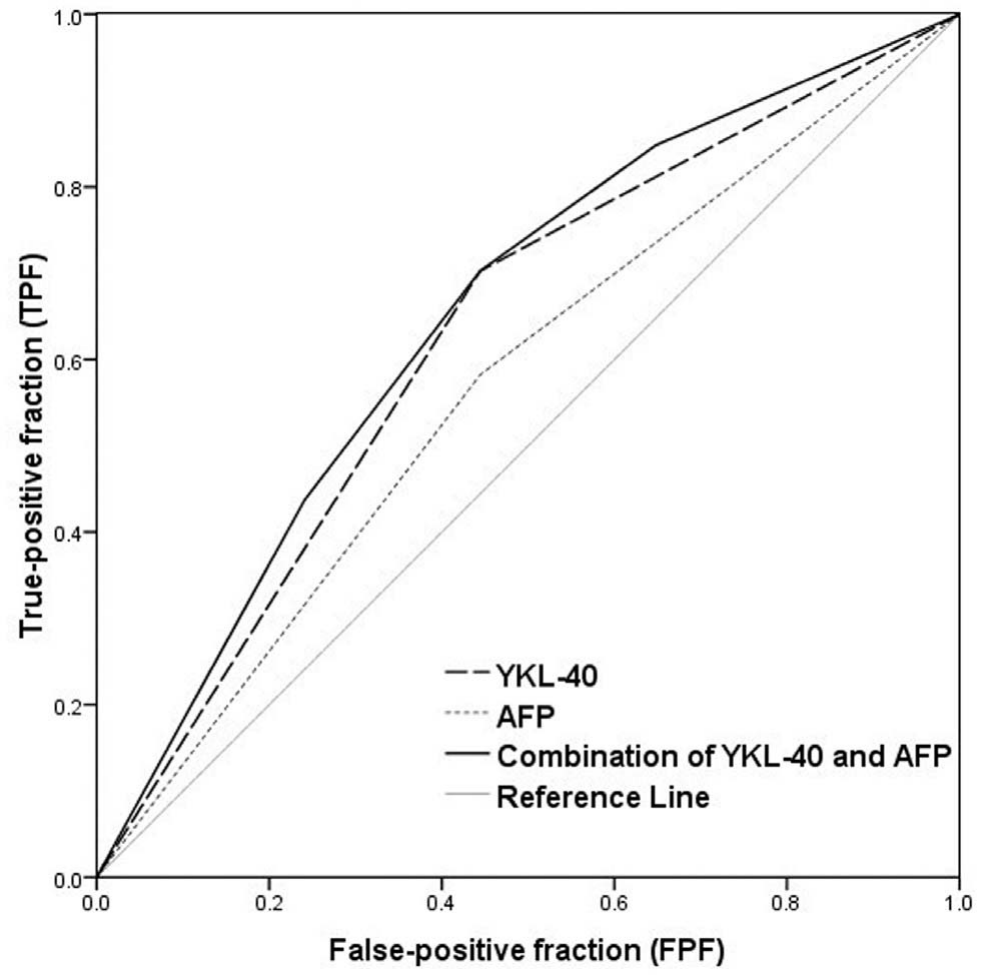
ROC curves for the mortality prediction by dichotomized YKL-40 and dichotomized AFP. The area under the curve was 0.629 for YKL-40, 0.569 for AFP and 0.652 for the combined YKL-40 and AFP.

Although serum AFP was not independently related to outcomes in the present study (Fig. 1b and Table 2), serum AFP was the common candidate for survival prediction of HCC patients. We further assessed the predictive values of dichotomized YKL-40 and dichotomized AFP for the mortality by receiver-operating characteristic curve analysis (Fig. 3 and Table 3). The area under the curve (AUC) of YKL-40 was 0.629 (95%CI: 0.541–0.717), which was larger than that of AFP (AUC = 0.569; 95%CI: 0.480–0.658), indicating that YKL-40 was superior to AFP in survival prediction. When serum YKL-40 and AFP were taken into consideration together, AUC of the combined YKL-40 and AFP was 0.652 (95%CI: 0.565–0.738), which was larger than that of YKL-40 and AFP. This showed that the combined YKL-40 and AFP improved the predictive sensitivity of survival prediction.

**Table 3:** Predictive values of dichotomized YKL-40 and dichotomized AFP for the mortality.

Serum marker	AUC	95%CI	*P* value
AFP	0.569	0.480–0.658	0.131
YKL-40	0.629	0.541–0.717	0.005
Combination ofYKL-40 and AFP	0.652	0.565–0.738	0.001

AFP, alpha-fetoprotein; AUC Area under curve; CI: confidence interval.

## Discussion

YKL-40 is not a cancer-specific biomarker. Elevated serum YKL-40 is found in a subset of patients with nonmalignant diseases characterized by inﬂammation and/or tissue remodeling [19]. Previous study has demonstrated that serum YKL-40 level has little utility as a cross-sectional screening tool for hepatobiliary malignancies [20]. Furthermore, serum YKL-40 may not serve as a serum biomarker for HCC because it cannot distinguish HCC from cirrhosis [13]. However, YKL-40 is an independent prognostic biomarker associated with a poorer clinical outcome of poor survival in genetically defined subgroups of different tumors. A recent study has showed that elevated YKL-40 predicts increased risk and poor survival of gastrointestinal cancer [15]. Most recently, a previous study from our group has demonstrated that serum YKL-40 was an independent unfavorable prognostic factor in HCC patients undergoing curative resection [16]. This prompted us to investigate the prognostic value of serum YKL-40 in HCC patients treated with TACE.

In this study, we found that serum YKL-40 was significantly elevated than the level in HCC patients compared to healthy controls *(P*<0.001),which provide additional evidence that elevated serum YKL-40 may play a role in cancer cell proliferation, survival, invasiveness and in the regulation of cell-matrix interactions [21]. Although it has been suggested that YKL-40 is expressed and secreted by macrophages, neutrophils, fibroblast-like synovial cells, chondrocytes, vascular smooth muscle cells and hepatic stellate cells [22], which may partly explain the elevation of serum YKL-40 in HCC patients, the exact mechanisms responsible for this abnormal secretion should be examined in vitro or in vivo in future study. In addition, serum YKL-40 was not associated with gender, etiology, Child-Pugh class, tumor size, vein invasion and number of TACE sessions, indicating that serum YKL-40 reﬂects other aspects of tumor growth, invasion and metastasis.

The current study showed that serum YKL-40 level was related to poorer overall survival in HCC patients undergoing TACE, whether it is evaluated continuously (*P* = 0.001) or dichotomously (*P*<0.001). Multivariate Cox regression analysis indicated that serum YKL-40 was an independent prognostic factor for OS in HCC patients (*P* = 0.001). This is in accordance with our previous study on serum YKL-40 in HCC patients undergoing curative resection [16]. Importantly, we did not observe any important prognostic impact of serum AFP on overall survival of HCC patients undergoing TACE, while in subgroups of low or high AFP level, serum YKL-40 still had the ability to discriminate patients with good prognoses from those with poor outcomes. When the predictive values of YKL-40 and AFP were studied by receiver-operating characteristic curve analysis, dichotomized YKL-40 was superior to dichotomized AFP in overall survival prediction. Our result suggests that serum YKL-40 may be a surrogate biomarker for prognostic prediction in HCC patients treated by TACE, especially in those patients with normal serum AFP levels.

AFP is the most widely used serum biomarker to evaluate the prognosis of HCC patients in clinical practice. Moreover, AFP remains the best marker to predict the recurrence and metastasis in AFP-positive HCC patients after operation [23],and recent studies showed that AFP have prognostic and predictive impact if determined prior to TACE [8]. However, in our present study, it was not independently related to outcomes, which may be explained by the possible reason that TACE treatment for HCC is not a radical cure. To consider the impact of serum AFP on OS as much as possible, YKL-40 and AFP were taken into consideration together. We found that the OS rates were significantly higher in the normal YKL-40 and low AFP group compared with the elevated YKL-40 and/or high AFP level group, and the predictive capacity of combination of serum YKL-40 and AFP was improved as shown by ROC analysis. This demonstrated that the combination of serum YKL-40 and AFP had more capacity to predict patients’ outcomes.

Additionally, our unvariate analyses showed that tumor size, vein invasion, and number of TACE sessions were also significant prognosis factors associated with OS of HCC patients undergoing TACE, but multivariate Cox regression analysis demonstrated that only tumor size was an independent prognostic factor for overall survival. This is not compatible with our previous report in HCC patients undergoing curative resection [16]. The reason is probably because of the difference in patient population, resectable HCC vs unresectable HCC.

Limitations of the study should be discussed. First of all, only HCC patients undergoing TACE were included in the present study. Secondly, this study is retrospective in design, and serum samples were not originally collected with the intent of testing the prognostic value of YKL-40. Thirdly, the follow-up samples after TACE were not collected, which make it not possible to assess the impact of post-TACE serum YKL-40 on overall survival. Therefore, our results need confirmation in an independent study.

In addition, microarray gene analyses and immunohistochemistry analyses mentioned in introduction are qualitative or semi-quantitative detection methods, which are mainly used for detection of YKL-40 expression in tissues and cells, while most serum analysis of YKL-40 are used ELISA method as we did, which is a quantitative detection method, and the YKL-40 ELISA has a low long-term CV. These characteristics make the results reliable and the analysis attractive in the clinical setting.

In conclusion, elevated serum YKL-40 level is a promising and feasible prognostic factor for the HCC patients treated with TACE. The underlying mechanism, which may help to develop a novel strategy to further improve the effect of TACE for HCC patients, still needs to be further investigated.
